# Psychometrics of MOOCs: Measuring Learners’ Proficiency

**DOI:** 10.5334/pb.515

**Published:** 2020-05-22

**Authors:** Dmitry Abbakumov, Piet Desmet, Wim Van den Noortgate

**Affiliations:** 1 ITEC, IMEC research group at KU Leuven, Kortrijk, BE; 2 Faculty of Psychology and Educational Sciences, KU Leuven, Leuven, BE; 3 eLearning Office, National Research University Higher School of Economics, Moscow, RU; 4 Faculty of Arts, KU Leuven, Leuven, BE

**Keywords:** Psychometrics, Item Response Theory, Massive Open Online Courses, Learning Analytics

## Abstract

Massive open online courses (MOOCs) generate learners’ performance data that can be used to understand learners’ proficiency and to improve their efficiency. However, the approaches currently used, such as assessing the proportion of correct responses in assessments, are oversimplified and may lead to poor conclusions and decisions because they do not account for additional information on learner, content, and context. There is a need for theoretically grounded data-driven explainable educational measurement approaches for MOOCs. In this conceptual paper, we try to establish a connection between psychometrics, a scientific discipline concerned with techniques for educational and psychological measurement, and MOOCs. First, we describe general principles of traditional measurement of learners’ proficiency in education. Second, we discuss qualities of MOOCs which hamper direct application of approaches based on these general principles. Third, we discuss recent developments in measuring proficiency that may be relevant for analyzing MOOC data. Finally, we draw directions in psychometric modeling that might be interesting for future MOOC research.

Massive open online courses (MOOCs) are “one of the most significant technological developments in higher education in the past decade” (Deng, Benckendorff, & Gannaway, 2019, p. 48). MOOCs are large scale web-based courses developed by universities, solely or in cooperation with industrial partners (for example, Coursera, 2015), in which anyone with internet access can participate. MOOCs are published on provider platforms, for instance, Coursera, edX, XuetangX, FutureLearn, Udacity, MiriadaX. MOOCs are a relatively new instructional form – the term did not exist before 2008 (Major & Blackmon, 2016). However, their popularity grows fast – over a decade more than 800 universities offered to learners more than 9,400 MOOCs (Shah, 2018a). In 2018 the largest MOOC provider, Coursera, achieved the milestones of 36 million registered learners and 3,000 courses (Shah, 2018b).

We consider courses from the world’s largest MOOC provider (Shah, 2019), Coursera, as typical MOOCs. These courses are composed of modules, each of which lasts a week and is structured around a single topic (Coursera, n.d.a.). Modules consist of smaller units, lessons. A lesson is structured around one or two learning objectives within a module topic. Each lesson includes a set of video lectures, reading assignments and formative assessments. A video lecture lasts 4–9 minutes and is accompanied by one or a set of formative assessment items that are incorporated in the lecture. It takes a student about 30 minutes to complete a lesson. Weekly modules are concluded by summative assessment, which is realized via a test, a peer-review task, or a combination of them. However, there are differences between courses, for instance, in length, workload, or options for self-paced learning.

Learners, professors, and universities – the key partners involved in MOOCs, – have an interest in accurate learners’ proficiency measuring. Learners take an online course and want to study efficiently. Proficiency measuring specifies learner’s position on the course-line, helps him/her to identify his/her strong and weak points and map areas that need additional work. Professors and their teams develop and optimize the course content. Here, the aggregated proficiency measures show to what degree the content incites learning and suggest improvements of video lectures, practical tasks, and support materials. Finally, universities award online course certificates to learners. The use of proficiency measures can provide evidence on whether and to what degree learners have mastered the course.

The learners’ proficiency is a latent construct; its measuring is a key concern of a scientific discipline within behavioral sciences – psychometrics (Borsboom & Molenaar, 2015). Latent constructs cannot be observed or measured directly. To get the estimates of proficiency, psychometricians need observable indicators of proficiency and a set of rules for linking the observable side to the latent construct. The typical indicators are learners’ responses on assessments, while the rules are provided by psychometric theories and statistical models.

The rapid development and expansion of MOOCs resulted in a growing body of related research (see the structured reviews of Bozkurt, Keskin, and de Waard (2016), Ebben and Murphy (2014), Liyanagunawardena and Williams (2013), Raffaghelli, Cucchiara, and Persico (2015), and Veletsianos and Shepherdson (2015) to get an overview of trends, topics, and methodology). The empirical research, in particular, is oriented at the challenges of teaching and learning (Deng, Benckendorff, & Gannaway, 2019), the motivation of both learners and teachers (Hew & Cheung, 2014), learners’ experience (Veletsianos, Collier, & Schneider, 2015). Surprisingly, although the aim of MOOCs is learning, in other words, a growth of learners’ proficiency, and MOOC platforms state that enhancing learning is a key focus (edX, n.d.; Coursera, n.d.b.), there is a lack of studies developing or using psychometric techniques in MOOC research. For instance, MOOC researchers and learning analytics still use grades, the simple proportion of correctly solved items in assessments or the (cumulative) proportion of assessments completed in a course, as a proxy for learners’ proficiency (for example, de Barba, Kennedy, & Ainley, 2016; Guo & Reinecke, 2014; Phan, McNeil, & Robin, 2016), which are simple and intuitive approaches but the use of them has a risk of bias due to oversimplification and ignoring factors of learners, content, and context. Abbakumov, Desmet, and Van den Noortgate (2018) mentioned that a Science Direct search revealed no papers for the combination of ‘MOOC’ and ‘psychometrics’ (or related keywords), the field within behavioral sciences focused on measurement. Finally, concluding a recently published review, Deng, Benckendorf, and Gannaway (2019) stated that MOOC research needs “theoretically driven, psychometrically sound” instruments.

We find imprecise or biased measures hamper the improvement and development of MOOCs and believe it is important to connect psychometric approaches and MOOC research. In this conceptual paper, we try to establish such a connection. First, we describe the principles of traditional approaches for measuring learners’ proficiency in education. Second, we discuss the qualities of MOOCs which hamper a direct application of these approaches based on the general principles in MOOCs. Third, we discuss recently developed solutions and potentially applicable approaches for measuring proficiency. Finally, we draw directions in psychometric modeling that might be interesting for future MOOC research.

## General Principles for Measuring Proficiency

There are two common theories in psychometrics – classical test theory and item response theory.

### Classical Test Theory

In 1888, Edgeworth suggested to decompose observed test scores into a true score and an error component (Edgeworth, 1888). Using an example on the evaluation of person essays, he stated that the mean judgment of competent raters represents the true score and deviations from that represent errors (Borsboom, 2005). Later this suggestion was elaborated into a theory which conceptualizes proficiency through the true score concept and now is known as the classical test theory (CTT; Lord & Novick, 1968; Novick, 1966), although the true score is not considered directly as latent in the theory. The respective classical test model:

1 {Y_j} = {\theta _j} + {\varepsilon _j},

is the most famous equation in educational and psychological measurement (Borsboom, 2005). According to this model, the test score of the *j*’th person (*Y_j_*) is the result of his/her proficiency (θ*_j_*) with a random measurement error (ɛ*_j_*). The error term (ɛ*_j_*) has an expected value of zero, and is assumed normally distributed, unrelated to the proficiency: E({\varepsilon _j}) = 0,{\rm{ }}{\varepsilon _j} \sim N(0,\sigma _\varepsilon ^2), and ρ_ɛθ_ = 0. Thus, the expected value of *Y_j_, E*(*Y_j_*), is θ*_j_*. As a result, when persons are given multiple tests measuring the same proficiency, the average score is randomly distributed around θ*_j_* with variance \sigma _\varepsilon ^2/n with *n* being the number of observations. Hence, the more observations, the closer the average score is in general to the proficiency.

The classical test model is simple for understanding which explains the high popularity of CTT among educational scholars and psychologists. At the same time, the simplicity leads to critical disadvantages. First, proficiency measures conceptualized through the test scores have a highly restricted area of generalization (Borsboom, 2005): conclusions are limited to the test itself or to an equivalent form, both statistically and in content domain, which is hard to find in practice. Second, proficiency measures are dependent on test difficulty: when the test is difficult, the person receives a low estimate of proficiency and when the test is easy, the person receives a high estimate of proficiency (Hambleton & Jones, 1993; Kean & Reilly, 2014; Kline, 2005). In practice, it means we cannot be confident about the proficiency measures’ comparability, even in case of replacement of a single item in a test. This is not only because these tests might not be equal in difficulty, they can also be different in content. Thus, proficiency measures conceptualized through the same test scores might not reflect the same proficiency in a content domain.

### Item Response Theory

Item response theory (IRT; Lord, 1952; Rasch, 1960; Birnbaum, 1968; van der Linden, 2016; Hambleton, Swaminathan & Rogers, 1991) was proposed as an alternative to CTT. The main idea of IRT is that a latent construct is considered to be unobserved determinant of a set of observed scores. For instance, a researcher who views proficiency in a specific domain as a latent variable assumes that the proficiency is the common cause of the person’s responses to a set of specific test items. IRT presents a broad class of models with nonlinear linking between person’s item responses (observable side) and his/her proficiency (latent construct). In the basic IRT model, the Rasch model (Rasch, 1960),

2 \begin{array}{l}
Logit\left({{\pi _{ij}}} \right) = {\rm{ln}}\left({\frac{{{\pi _{ij}}}}{{1 - {\pi _{ij}}}}} \right) = {\theta _j} - {\delta _i}\;and\;\\
{Y_{ij}} \sim Bernoulli\left({{\pi _{ij}}} \right),
\end{array}

the probability (π*_ij_*) of the correct response of person *_j_* to the item *i* is described by a logistic function of the difference between the person’s proficiency parameter (θ*_j_*) and the item difficulty parameter (δ*_i_*). To fit the Rasch model, a marginal maximum likelihood procedure is often used to estimate the item difficulty parameters (Bock & Aitkin, 1981), assuming that the persons are a random sample from a population in which the person proficiencies are normally distributed with {\theta _j} \sim N(0,\sigma _\theta ^2), while the items have fixed difficulty. Individual person parameters can be estimated afterwards using empirical Bayes procedures (Van den Noortgate, De Boeck, & Meulders, 2003). Later this measurement model was extended to model complex observed variables (for instance, Bock, 1972; Samejima, 1969; Thissen & Steinberg, 1984) using complex latent constructs (for instance, Adams, Wilson, & Wang, 1997; Embretson, 1980; Maris, 1995; Whitely, 1980).

In comparison to CTT, person’s proficiency parameters in IRT are independent of the test difficulty. It allows comparing persons even in case of partial replacement of items. However, IRT is demanding in terms of required sample sizes to obtain stable parameter estimates. For instance, Hambleton and Jones (1993) suggest to use a minimum of 500 persons to fit the model.

## Issues on Measuring Proficiency in MOOCs

The key issues which hamper the direct use of the common psychometric techniques in MOOCs are linked to understanding the concept of proficiency itself.

First, both CTT and IRT assume the proficiency does not change within a test (Lord & Novick, 1968). This way of understanding critically contrasts to the reality of processes in MOOCs and any learning process where the main product is the change of proficiency. MOOCs generate a change in proficiency at multiple levels. The first level is the level of assessment. Typically, a MOOC learner is allowed to use several (even an infinite number of) attempts to solve assessment tasks (Coursera, n.d.c.). If the learner fails at one attempt, he/she can be provided with help information, review a video lecture, use external materials, and then make a new attempt. Thus, the learner’s proficiency may change with each new attempt to solve the certain task or the assessment in a whole. The second level is the level of a course. A MOOC learner watches video lectures, practices with formative assessments, discusses problems on a course forum. These activities are the main source of learning. Obviously, the learner’s proficiency is not the same in two certain points of a course. Therefore, we cannot neglect the dynamic character of proficiency while estimating it.

Second, IRT assumes that proficiency (or a set of proficiencies) is a common cause of the learners’ responses. However, MOOC learners have a high degree of freedom because of low-stakes of such courses, especially in case of low or no integration in a curriculum. Online learners’ performance and retention are linked to a number of unrelated to knowledge emotional and motivational characteristics (de Barba, Kennedy, & Ainley, 2016; Hart, 2012; Hew & Cheung, 2014). Thus, the learner’s proficiency might not be a single determinant of correctness in his/her responses in MOOCs. Therefore, neglecting the importance of such factors in performance explanation might result in biased conclusions on proficiency – learners may perform weakly, not because they have low proficiency, but rather because they experience the lack of motivation.

At the same time there are at least two specific issues related to the observable side, indicators of proficiency, in MOOCs. First, changes in tests are relatively frequent in MOOCs – professors often replace or add new items on the fly. This is a critical limitation for CTT as discussed above, but also induced additional complexity for using IRT as the difficulty of new items typically is not known. A second issue is that IRT requires a relatively large number of items in assessments to provide accurate proficiency measures. Kruyen, Emons, and Sijtsma (2012) stated that the minimally required test length for individual level decisions is 40 items. By contrast, MOOCs often offer 15 or even fewer items in summative assessments which is a significant constraint for direct use of IRT. In addition, although the sample size issue might hamper the use of IRT in MOOCs due to the number of learners drops quickly, taking into account that the average number of MOOC enrollers is 43,000 and the average course completion rate is around 10% (Ferenstein, 2014), most courses meet the IRT requirement to use a minimum of 500 persons to fit the model (Hambleton & Jones, 1993).

As can be seen from the above, the common psychometric models of CTT and IRT are not tailored to use directly for measuring proficiency in MOOCs and should be tuned up accordingly.

## Current Developments on Measuring Proficiency in MOOCs

Recently, several extensions of the Rasch model were proposed for modeling the learners’ performance in MOOCs. These include extensions for modeling the dynamics in proficiency on multiple levels (Abbakumov, Desmet, & Van den Noortgate, 2018; 2019) and the inclusion of additional latent variables (Abbakumov, Desmet, & Van den Noortgate, in press).

### General IRT Framework

Extending the Rasch model without running into computational issues of model overidentification became possible by the use of the reformulation of the Rasch model proposed by Van den Noortgate, De Boeck, and Meulders (2003). In this reformulation based on the principle of cross-classification multilevel models, we have the intercept, and two residual terms referring to the person *j* and the item *i* respectively:

3 Logit\left({{\pi _{ij}}} \right) = {b_0} + {u_{1j}} + {u_{2i}},

where {u_{1j}} \sim N(0,\sigma _{u1}^2)\ {\rm{ and\ }}{u_{2i}} \sim N(0,\sigma _{u2}^2), and *Y_ij_~Bernoulli*(*π_ij_*). As the mean of both residual terms equals zero, the intercept corresponds to the estimated logit of the probability of the correct response for an average person on an average item. The first residual term, *u_1j_*, this residual term can be interpreted as the proficiency of person shows the deviation of the expected logit from person *j* from the mean logit. The higher this deviation, the higher the expected performance. In terms of the original formulation of the Rasch model from Equation 2, this residual term can be interpreted as the proficiency of person *j*, and is equivalent to *θ_j_*. The second residual term shows the deviation of the expected logit from item *i* from the overall logit. Here, the larger the residual – the higher the probability of the correct response is. In that sense, the residual term *u_2i_* presents the relative difficulty of item *i*, compared to the mean item difficulty, *b_0_*. Therefore, the difficulty parameter *δ_i_* from the original formulation of the Rasch model is equivalent to –(*b_0_* + *u_2i_*) in the reformulation from Equation 2. Thus considering both items and persons as random leaves degrees of freedom to estimate the effect of predictors. The general principles of extending the Rasch model and other psychometric models by including explanatory predictors might be found in the work of De Boeck and Wilson (2004) and collaborators.

### Proficiency Dynamics within the Assessment

As we mentioned above, the key assumption of IRT is that the proficiency does not change within a test (Lord & Novick, 1968). In MOOCs this assumption is met only partly. We may indeed expect that the learner’s proficiency remains constant within particular summative assessment which typically does not lasts longer than 30–45 minutes. However, if the learner takes several attempts, the chances for the correct response grow with each new attempt. The first reason for this is learning. For instance, MOOCs typically provide learners with hints in case of a wrong response. This instructional content is aimed at helping the learner to understand his/her mistake, guiding through relevant learning materials and preparing for the next attempt. The second reason is that sometimes the learner may simply enumerate possibilities, for example, by clicking repeatedly on alternative options in multiple-choice questions.

Abbakumov, Desmet, and Van den Noortgate (2018) proposed to consider two components of proficiency within weekly summative assessment in a MOOC – a constant and a dynamic component, – and introduced the following model for learners’ performance:

4 \begin{array}{l}
Logit\left({{\pi _{ij}}} \right) = {b_0} + ({b_{10}} + {b_{1j}})*attemp{t_{ij}}\\
+ {u_{1j}} + {u_{2i}}\;and\;{Y_{ij}} \sim Bernoulli\left({{\pi _{ij}}} \right),
\end{array}

where *b_0_* equals the estimated logit of probability of the correct response of an average student on an average item in weekly summative assessment; *attempt_ij_* is 0, 1, 2, 3 or 4 and means the first, the second, the third, the fourth, or the fifth or higher attempt respectively; *b_10_* is overall effect of attempt, while {b_{1j}} \sim N(0,\sigma _{b1}^2) is a deviation of the attempt effect for student *j* from the overall effect; and {u_{1j}} \sim N(0,\sigma _{u1}^2{\rm{)\ and\ }}{u_{2i}} \sim N(0,\sigma _{u2}^2). Thus, the random deviation *u_1j_* can be interpreted as the proficiency of learner *j*, which is hardly changing within summative assessment, and is equivalent to *θ_j_* in the Rasch model from Equation 2, while the dynamic component (*b*_10_ + b_1j_)*attempt_*ij*_ shows learner’s *j* individual increase in chances to solve the item *i* correctly with a new attempt which can be interpreted as learner’s *j* local learning about item *i* which came with the use of the instructional content associated to item *i* or just repeated guessing. The authors showed that learners who use a higher number of attempts have lower ‘local learning’ (this is the increase of the chance on a correct answer with an additional attempt), which can help to distinguish between learners who learn and who use attempts to enumerate the item options. The researchers also noted that the effect of an additional attempt may vary from item to item, for example, a multiple-choice item with four options could be solved correctly (using simple enumeration) by four attempts maximum, while for solving an open-ended item, where the student should indicate a number or a word, the number of attempts may be much higher. In order to account for this variation, they proposed the following composition of the dynamic component (*b*_10_ + b_1*j*_ + b_1*i*_)* attempt_*ij*_, where {b_{1i}} \sim N(0,\sigma _{b1}^2) is a deviation of the attempt effect for item *i* from the overall effect. Finally, the authors showed that learners who perform better in practice and more active with watching video lectures have higher chances to solve summative assessment items correctly.

It is worth to mention that including all learners’ responses into analysis, while accounting for the number of attempts, gives an unbiased view on the proficiencies and moreover allows to study the evolution of the performance over attempts. In contrast, analysis of all responses without accounting for the number of attempts would obscure real differences between students in their proficiency, while including only the scores at the first attempt, would reduce the amount of information used and hence decrease the accuracy of estimates and the power of statistical tests. The cross-validation on the data from three MOOCs from the Coursera platform revealed 6% improvement in accuracy of predicting the correctness of learners’ responses on summative assessment items for the extended model in comparison to the traditional Rasch model (Abbakumov, Desmet, & Van den Noortgate, 2018). These improvements show that including learners’ responses with accounting for the number of attempts to the analyses gives a more accurate view of the learners’ proficiencies.

### Proficiency Dynamics through the Course

Another type of change in learners’ proficiency in MOOCs which is not accounted for in the common psychometric models is growth through the course. Taking into account that video lectures are the central instructional tool in MOOCs (Coursera, n.d.d.), researchers proposed to measure the growth via the estimation of individual effect of the cumulative sum of video lectures a learner watched before a certain assessment task on the correctness of his/her response this task in a MOOC (Abbakumov, Desmet, & Van den Noortgate, 2019). Thus the growth in learners’ proficiency from video lectures is considered as the growth through the course.

In the proposed Rasch model extension,

5 \begin{array}{l}
Logit\left({{\pi _{ij}}} \right) = {b_0} + ({b_{10}} + {b_{1j}})*vide{o_{ij}} + \ldots \\
+ {u_{1j}} + {u_{2i}}\;and\;{Y_{ij}} \sim Bernoulli\left({{\pi _{ij}}} \right),
\end{array}

where *b_10_*, this is the effect of the progressive sum of videos looked at, can be interpreted as the overall growth through the course, while b1j is the deviation of the progressive sum effect for student *j* from the overall effect, interpreted as the deviation of the individual growth through a course from the overall growth over subjects; *u*_1*j*_ and b_1*j*_ are assumed to follow univariate normal distributions, N(0,\sigma _{u1}^2),{\rm{ }}N(0,\sigma _{b1}^2), or a multivariate normal distribution *N*(0,Σ) with Σ as the variance-covariance matrix. The value of *u*_1*j*_ can be considered as the initial proficiency of learner *j* (*θ*_0*j*_), while the value *u*_1*j*_ + (*b*_10_ + *b*_1*j*_)* *video_ij_* corresponds to the proficiency of learner *j* at the moment of responding on item *i* (*θ_ij_*), when the number of videos looked at by learner *j* equals *video_ij_*.

The use of the extension showed that the probability of the correct response grows with every new watched lecture and the growth effect is specific for individual learners – for some learners, the growth may be intensive, while for some learners it may be almost flat through the whole course. In the cross-validation study, the quality of predicting correctness of learners’ responses on summative assessment items tested on the data from three MOOCs from the Coursera platform improves with 3.3% while using the extension in comparison to the use of original Rasch model. This fact promotes the use of extensions as a better approach in measuring the learners’ proficiency and its growth in MOOCs.

A complementary solution can be adapted from educational online games. Researchers proposed an IRT model (Kadengye, Ceulemans, and Van den Noortgate, 2014; 2015) where learners’ proficiency is considered as a function of time within learning sessions and the time between learning sessions:

6 \begin{array}{l}
Logit\left({{\pi _{ij}}} \right) = \left({{\alpha _0} + {\omega _{0j}}} \right) + \left({{\alpha _1} + {\omega _{1j}}} \right)\\
*wtim{e_{ij}} + \left({{\alpha _2} + {\omega _{2j}}} \right)*btim{e_{ij}} + {v_i}\;\\
and\;{Y_{ij}} \sim Bernoulli\left({{\pi _{ij}}} \right),
\end{array}

where *α*_0_ is the overall initial learners’ proficiency, *ω*_0*j*_ is the deviation of the initial proficiency of learner *j* from *α*_0_, *wtime_ij_* and *btime_ij_* is the amount of time that passed for learner *j* while respectively using and not using the learning environment, up to the moment learner *j*’s response to item *i, α*_1_ and *α*_2_ are overall population linear time trends within and between sessions respectively, and *ω*_1*j*_ and *ω*_2*j*_ are deviations of the time trends from learner *j* from *α*_1_ and *α*_2_ respectively. The learner-specific random effects are assumed to have a multivariate normal distribution, and *vi* are random item effects with {v_i} \sim N(0,\sigma _v^2). Thus, the authors introduce a dynamic concept *θ_ij_* = (*α*_0_ + *ω*_0*j*_) + (*α*_1_ + *ω*_1*j*_)*wtime_ij_ + (*α*_2_ + *ω*_2*j*_)*btime_ij_, which corresponds to the proficiency of learner *j* at the moment of responding item *i*. However, to apply this solution in MOOCs, the platform has to log the time a learner works with an online course. This is not yet a common practice.

### Additional Latent Effects on the Performance

The first additional latent effect on the learners’ performance in MOOCs which was tested is interest (Abbakumov, Desmet, & Van den Noortgate, 2020). Interest has been chosen because it is a “critical cognitive and affective motivational variable” (Renninger & Hidi, 2011, p. 169), which improves learners’ performance in different domains. The researchers decided to test the effect of interest in formative assessments (ungraded practice assessments that guide and support learning) in MOOCs because in comparison to summative assessments (graded assessments that measure progress toward learning objectives) these items are incorporated into video lectures, thus, learners’ performance might not only be determined by proficiency, but also by learners’ interest to the specific video lecture.

The researchers proposed the following extension:

7 \begin{array}{l}
Logit\left({{\pi _{ij}}} \right) = {b_0} + ({b_{10}} + {b_{1j}})*interes{t_{ij}}\\
+ \ldots + {u_{1j}} + {u_{2i}}\;and\;{Y_{ij}} \sim Bernoulli\left({{\pi _{ij}}} \right),
\end{array}

where *interest_ij_* reflects learner’s *j* interest to the video lecture in which the formative assessment item *i* is incorporated in terms of his/her response on a question “Please rate the level of your interest during the video” with five Likert-type options: “very high”, “high”, “neutral”, “low”, and “very low” which are scored as 0, –1, –2, –3, –4 respectively; *b*_0_ equals the estimated logit of the probability of the correct response of an average learner to an average formative assessment item incorporated into the video lecture of the course in case of very high reported interest; *b*_10_ reflects the overall effect of interest on the expected performance, this is the expected increase of the logit when interest increases with one unit; however, the effect of interest may not be the same for all learners, thus, to model such individual differences, the researchers used a random deviation of the interest effect for learner *j* from the overall effect, and {b_{1j}} \sim N(0,\sigma _{b2}^2); and {u_{1j}} \sim N(0,\sigma _{u1}^2) and {u_{2i}} \sim N(0,\sigma _{u2}^2).

As a product of applying this extension an interesting finding was found where the intercept variance, this is the variance between students in the effect of proficiency, was reduced by 25% by including a random interest effect. This fact provides a more nuanced insight in the role of proficiency on the learners’ performance and confirms the importance of taking interest into account. However, there was no significant improvement in response prediction accuracy found compared to a model not taking into account interest.

## Further Directions in Measuring Proficiency in MOOCs

In this section we highlight a set of promising directions for further development of psychometrics of MOOCs: the measurement of complex outcomes and latent constructs, the tracking learners’ progress on-the-fly, the improved understanding learners’ performance by the use of explanatory psychometric modeling approaches, the advancement in the quality of predictions by increasing the model complexity, the synergy of different psychometric methods and their combination with machine learning for precise and interpretable conclusions on learners.

### Measuring Complex Outcomes and Latent Constructs

Learners interact with MOOC content in different ways: they watch video lectures, read PDF assignments, discuss on forums, they attempt solving assessments. All these activities are interlaced and result in complex outcomes, for instance, a partly correct response made with a hint, after re-watching the video lecture and after discussing on the forum. To have a more nuanced view on learners’ proficiency, in this case a researcher should consider extending a model from polytomous IRT family (see Ostini & Nering (2005) for an overview) which model outcomes scaled in more than two categories (for example, “correct/partially correct/wrong” instead of dichotomous “correct/wrong”). Moreover, a researcher should consider including predictors describing learners’ activity before his/her response alongside as well as their interaction.

MOOCs use not only test-based assessments. An important type of assessment is peer-reviewed assignments. In such assignments a learner’s work is generally assessed by at least three peers using a schema provided by a course professor. An important problem of such assessments is a lower precision or validity due to peers’ subjectivism (Admiraal, Huisman, & van de Ven, 2014; Kravchenko, 2018). For instance, a learner may get low score not because of his/her low proficiency, but because of high severity of the peer who assessed the work. In order to assess and correct for bias that arose by peers’ leniency or severity, a researcher should consider tuning up models which look at a learner’s score as a common effect of his/her proficiency, difficulty of the task, and raters’ effect. These models are well developed in IRT framework (Linacre, 1992; Myford & Wolfe, 2003; Myford & Wolfe, 2004).

MOOCs combine multiple domains within one course – one course may form a number of skills. Learners’ responses in assessments might be caused by a set of proficiencies, for instance, to solve a specific task in bioinformatics a learner might need a knowledge in calculus, programming, biology. Thus, understanding a single proficiency as a common cause of this response seem to be oversimplified. In this case a researcher should consider a multidimensional solution (Reckase, 2009) which in general case replaces *θ_j_* in the original Rasch model from Equation 2 with Σ*_k_b_ik_θ_jk_* where *θ_jk_* is the proficiency of learner *j* on the *k*th dimension, and *b_ik_* is the factor loading of item *i* on dimension *k*. Another psychometric way to model complex latent constructs is to use cognitive diagnostic models (CDM; Bolt, 2007; Junker & Sijtsma, 2001; de la Torre, 2009). These models assume a latent cognitive profile that represents mastery status on a set of specific skills involved in answering an item. Thus, CDMs enable researchers to better understand each learner’s strengths and weaknesses in terms of each specific skill and support fine-grained formative feedback on each skill. An alternative to IRT and CDM approaches to deal with complex latent constructs is a Knowledge Space Theory approach (KST; Doignon & Falmagne, 1985). The KST assumes that every knowledge domain can be represented as a set of items and dependencies between these items in that knowledge of a given item (or a subset of items) may be a prerequisite for knowledge of another, more difficult or complex item. Thus, a researcher can estimate what a learner can do now and what a learner is ready to learn.

### Tracking the Progress On-the-Fly

The approaches from Equations 5 and 6 work for post-hoc measuring, not for dynamic growth tracking. The on-the-fly progress estimation, crucial for navigation and recommendations engines which decide about when to support a learner or to advance him/her through a course, could be realized by the use of the Elo Rating System (ERS; Elo, 1978). The ERS was initially developed for ranking chess players where the rank update is based on the weighted difference in match result and expected match result. The researchers (Brinkhuis & Maris, 2009; Klinkenberg, Straatemeier, & van der Maas, 2011) suggested to use the ERS to dynamically update learners’ proficiency based on the results of solving items in an online educational game. There the proficiency of learner *j* at the moment of responding item *i* is computed as {\hat \theta _{ij}} = {\hat \theta _{ij}} + K\{ {Y_{ij}} - E({Y_{ij}})\}, where *K* is the constant term presenting a step size in learners’ proficiency update, which in learning environments is typically set to be 0.4, *Y_ij_* is actual response of learner *j* on item *i* which is coded dichotomously, where 0 stands for an incorrect response and 1 stands for a correct response, and *E*(*Y_ij_*) is his/her expected response on this item. The expected response can be computed using the Rasch model from Equation 2, more specifically as a function of the difference between the ability as estimated before the response was given, {\hat \theta _{(i - 1)j}}, and the difficulty of the item, *δ*_i_. This approach is widely used in learning environments (Pelanek, 2016; Oefenweb, n.d.) and is potentially applicable in MOOCs.

An alternative modeling approach is Bayesian Knowledge Tracing (BKT; Corbett & Anderson, 1995) frequently used in the field of Intelligent Tutoring Systems (ITS; Psotka, Massey, & Mutter, 1988) for tracking the process of learners’ knowledge acquisition. The approach also serves as the basis for selecting the next skill that a learner should work on, once the current skill has been mastered. In BKT, skills are modeled as dichotomous variables (where 1 stands for mastered skill and 0 stands for not mastered skill) and learning is characterized as a transition between 0 and 1. The standard BKT model utilizes five global parameters per skill denoting the probability that a learner is in the mastery state for the skill before interacting with an item utilizing that skill, has mastered or has not mastered the skill or has guessed or has slept while interacting with an item utilizing that skill. Although the original BKT model does not permit learner-specific or item-specific parameters, a set of extensions has been developed, including effects of learners’ individual characteristics (Yudelson, Koedinger, & Gordon, 2013) and item difficulty (Pardos & Heffernan, 2011). In addition, there is a recent development on combining BKT and IRT frameworks (Deonovic, Yudelson, Bolsinova, Attali, & Maris, 2018).

### Explanatory Modeling

There are two general psychometric approaches that might be involved in work with learners’ responses on assessment items in MOOCs. The first type is the measurement approach. This approach seeks the optimal way of locating an individual learner on the latent scale, the scale of proficiency. In other words, a researcher tries to estimate an individual learner’s proficiency as precisely as possible, and all the techniques we discussed above belong to measurement approaches.

The second type is an explanatory approach. This approach is focused on explaining learners’ responses in terms of other variables. For instance, a MOOC researcher might be interested in studying the relationship between the learners’ performance and their previous learning experience to understand the optimal way to structure this experience in order to improve the performance. For instance, recent results of Abbakumov, Desmet, and Van den Noortgate (2018), showing that MOOC learners who are productive with formative assessments have higher chances to solve summative items correctly, may suggest us to try to intensify practice in courses. Another interest would be to explore design variations in the items compose the assessment, to see if performance on the items depends on, specific item features (for instance, multiple choice response or typed response) in order to optimize the assessment composition. As can be seen, the level of conclusions here is not the individual learner, but rather the general inferences that can be made about the relationship of explanatory variables across the learners and the items. Therefore, the explanatory approach might be very useful in A/B tests (large-scale online randomized controlled experiments) which seek the optimal way to organize learning experience within the digital environment, for instance, in comparing two types of video lectures – produced in professional studio (A) and hand-crafted (B) – by their effect on learners’ performance in a MOOC (Kizilcec & Brooks, 2017; Savi, Williams, Maris, & van der Maas, 2017).

The explanatory movement has been started by De Boeck and Wilson (2004) with collaborators. Although there are a set of models developed for different explanatory strategies, for instance, to explain item-related or person-related variance, we find the cross-classification multilevel logistic approach (Van den Noortgate, De Boeck, & Meulders, 2003) which combines the both strategies to be a universal and flexible framework a MOOC researcher might use. The two approaches, the measurement approach and the explanatory approach, can be combined within this framework.

### Improving Prediction

As we saw above, tuning the common psychometric models results in improvements in the accuracy of predicting learners’ responses. However, these improvements are rather small, for instance, 3–6% (Abbakumov, Desmet, & Van den Noortgate, 2018; 2019). Thus a researcher might be interested in better predictive capacity from psychometric solutions, for instance, accurate predictions on learners’ performance are necessary in building adaptive learning experience, for instance, in automated navigation through learning materials or a system of personalized hints and recommendations. There is substantive advancement in boosting predictive power in statistical learning theory, a framework for machine learning based on statistics, which deals with the problem of finding a predictive function based on data (Hastie, Tibshirani, & Friedman, 2009). In general, these improvements are linked to growing model complexity. A MOOC researcher should consider to move beyond linearity in order to improve the predictive power. To this end, a MOOC researcher can use several techniques, for instance, polynomial regression or regression splines. Polynomial regression extends the linear model by adding predictors, got by raising each of the original predictors to a power (for instance, a quadratic regression uses two variables, *X*, and *X^2^*, as predictors). This approach provides a simple way to provide a nonlinear fit to data. Regression splines are more flexible than polynomials. They split the range of a variable into *K* distinct intervals. Within each interval, a polynomial function is fit to the data. Thus, instead of fitting a high-degree polynomial over the entire range of *X*, piecewise polynomial regression involves fitting separate low-degree polynomials over different intervals of *X*. This can produce a very flexible fit. However, advancement in prediction might come alongside with significant decrease in model interpretability which can be a critical constraint for applications for educational purposes. The key focus of a MOOC researcher here is to find optimal quality of prediction without loss in understanding functional relationship between variables.

### Mixing Methods

The popular term “there is no free lunch in statistics” (Wolpert & Macready, 1997) which means no one method dominates others over all possible situations and data sets spreads on psychometrics of MOOCs as well. For example, the Rasch model extensions provide advantages in understanding learners’ performance in MOOCs, however, there still is a room in predictive power to fill in. Another example, ERS works well in updating learners’ proficiency in online educational games but may suffer from the cold-start problem, when the program does not know a new learner’s proficiency level at the beginning of the learning. These examples draw a perspective direction on mixing psychometric approaches and combining them with machine learning methods to cover gaps. For instance, to solve the cold start problem Park, Joo, Cornillie, van der Maas, and Van den Noortgate (2019) combine ERS and explanatory psychometric modeling. The same problem has been addressed in a work of Pliakos and colleagues (2019) but using a combination of IRT models and decision tree method from machine learning. As can be seen there are several alternative solutions for the same problem can be found.

There are a number of methods in machine learning a researcher may consider to combine with psychometric approaches, for instance, tree-based methods, support vector machines, clustering. Using these methods may result in dramatic improvements in the quality of conclusions although there is no guarantee of such improvements (Wilson, Karklin, Han, & Ekanadham, 2016). However, the cost for these improvements is a significant loss in interpretation, for instance, why in a given situation we have high or low performance (or high or low learning), which makes machine learning methods somewhat ‘black boxes’. At the same time, in comparison to many machine learning methods, a major advantage of psychometric models is their explanatory power: they give insight in what works, when and for whom, and therefore may help to further optimize MOOCs. Thus a researcher has to find an equilibrium in such combinations. In principal we agree with a direction on creating models that are interpretable in the first place which is growing up now in data science (Rudin, 2019).

## Conclusion

Psychometrics of MOOCs is a very recent development in the field. To find an answer on the question of when and why learning does happen in MOOCs, and how these digital learning products do work, it combines a century-old heritage of psychometrics and modern sources of the logged data. It has its unique properties, such as dynamic character of learners’ proficiency and composite character of cause of learners’ performance. Although it is already showing improvements in understanding the digital learners, its future is linked to moving towards computational direction involving complex data and advanced statistical procedures into modeling multidimensional dynamic constructs and processes in MOOCs.
